# Transgenic Mouse Models in Cancer Research

**DOI:** 10.3389/fonc.2018.00268

**Published:** 2018-07-20

**Authors:** Ursa Lampreht Tratar, Simon Horvat, Maja Cemazar

**Affiliations:** ^1^Department of Experimental Oncology, Institute of Oncology Ljubljana, Ljubljana, Slovenia; ^2^Biotechnical Faculty, University of Ljubljana, Ljubljana, Slovenia; ^3^Faculty of Health Sciences, University of Primorska, Isola, Slovenia

**Keywords:** transgenic mice, genetically engineered mouse models, patient-derived xenograft models, humanized mouse models, CRISPR/Cas9, non-germline genetically engineered mouse models

## Abstract

The use of existing mouse models in cancer research is of utmost importance as they aim to explore the casual link between candidate cancer genes and carcinogenesis as well as to provide models to develop and test new therapies. However, faster progress in translating mouse cancer model research into the clinic has been hampered due to the limitations of these models to better reflect the complexities of human tumors. Traditionally, immunocompetent and immunodeficient mice with syngeneic and xenografted tumors transplanted subcutaneously or orthotopically have been used. These models are still being widely employed for many different types of studies, in part due to their widespread availability and low cost. Other types of mouse models used in cancer research comprise transgenic mice in which oncogenes can be constitutively or conditionally expressed and tumor-suppressor genes silenced using conventional methods, such as retroviral infection, microinjection of DNA constructs, and the so-called “gene-targeted transgene” approach. These traditional transgenic models have been very important in studies of carcinogenesis and tumor pathogenesis, as well as in studies evaluating the development of resistance to therapy. Recently, the clustered regularly interspaced short palindromic repeats (CRISPR)-based genome editing approach has revolutionized the field of mouse cancer models and has had a profound and rapid impact on the development of more effective systems to study human cancers. The CRISPR/Cas9-based transgenic models have the capacity to engineer a wide spectrum of mutations found in human cancers and provide solutions to problems that were previously unsolvable. Recently, humanized mouse xenograft models that accept patient-derived xenografts and CD34+ cells were developed to better mimic tumor heterogeneity, the tumor microenvironment, and cross-talk between the tumor and stromal/immune cells. These features make them extremely valuable models for the evaluation of investigational cancer therapies, specifically new immunotherapies. Taken together, improvements in both the CRISPR/Cas9 system producing more valid mouse models and in the humanized mouse xenograft models resembling complex interactions between the tumor and its environment might represent one of the successful pathways to precise individualized cancer therapy, leading to improved cancer patient survival and quality of life.

## Introduction

The mouse as a model for human cancer research has proven to be a useful tool due to the relatively similar genomic and physiological characteristics of tumor biology between mice and humans. Mice have several similar anatomical, cellular, and molecular characteristics to humans that are known to have critical properties and functions in cancer. Additionally, the proportion of mouse genes with a human ortholog is 80% (1), thus providing an excellent experimentally tractable model system as a research tool to investigate basic mechanisms of cancer development as well as responses to treatment (2). Although conventional transgenic mouse models have remained a valuable tool to examine the molecular mechanisms of carcinogenesis, a limitation has been a low degree of heterogeneity in mouse tumors in comparison to the very heterogeneous human tumors. Several advances have been made in modeling cancer in mice, and the new models described in this review are now more capable of modeling human cancers with mutations that are controlled spatially and/or temporally. In addition, these models better address tumor heterogeneity and inter-patient variability in the clinical setting (3).

Traditionally, immunocompetent and immunodeficient mice with syngeneic and xenografted tumors transplanted subcutaneously or orthotopically have been used. These models are still widely applied for many different types of studies and are also affordable (4). However, in the early 1980s, new types of mouse models emerged with engineered mutations in their genome that revolutionized cancer research. In 1974, R. Jaenisch and B. Mintz performed an experiment wherein the viral oncogenes from simian virus (SV40) were microinjected into the blastocoel of mouse embryos. Although the resulting mice did not develop tumors, they could detect the viral DNA integrated in the genome of cells of many different tissues. These mice are considered the first transgenic mice (5). Later in the 1980s, the first transgenic mouse cancer models were produced, which were genetically engineered to express dominant oncogenes. With these so-called “oncomice,” the predispositions required for the development of cancer, as well as new targets for the development of novel therapeutic approaches, could be investigated. Later, more definitive modifications in the genome were performed with knockout and knock-in mice, and since then several research papers have used the term transgenic mice as a distinct group from knockout and knock-in mice (6). Due to the confusion related to the nomenclature, in 2007, the Federation of European Laboratory Animal Science Associations released guidelines for the production and nomenclature of transgenic rodents. In these guidelines, it is stated that transgenic animals are referred to as those with spontaneous, chemically induced mutations and those with random or gene-targeting DNA recombination events (7). The National Institute of Health, National Cancer Institute (NIH NCI) refers to the term transgenic animals for models in which DNA from the mouse genome or from the genome of another species has been incorporated into each cell of the mouse model genome (8). These mice are now mostly called transgenic mice, but they are also referred to as germline genetically engineered mouse models (GEMM), including knock-in and knockout mouse models (9). Additionally, the mouse genome informatics database (10), the main database resource for the laboratory mouse, lists mutant alleles for each gene in several categories, including “transgenic models” similar to the definition in reference 8 above and “targeted,” which encompasses both targeted knockout and knock-in models similar to the definition of GEMM above. In this article, we refer to the term “transgenic” as a general term for all types of genome alterations in the germ or somatic cells and/or *in vitro* and *in vivo* mouse models.

## Production of Transgenic Mice

**Figure 1:**
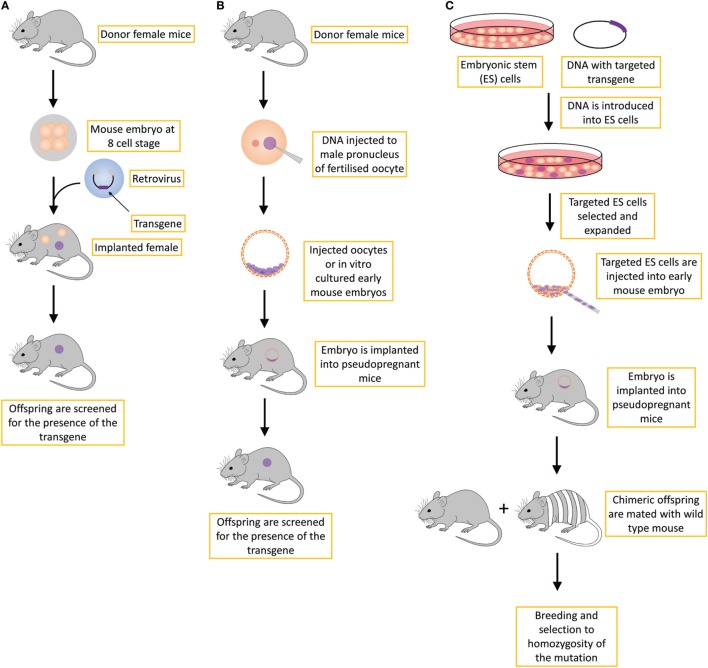
Different strategies for creating transgenic mice include the **(A)** retroviral approach, which is not routinely used; **(B)** standard transgene approach, in which the DNA is inserted into the genome in an unspecific manner; and **(C)** gene-targeted transgene approach, which is an approach that is routinely used to create conventional knockout transgenic mice, usually with a constitutive loss-of-function mutation.

Transgenic mice can be produced in several ways by introducing DNA into the mouse genome (Figure 1).

1. By retroviral infection of mouse embryos at different developmental stages. This method is not routinely used for the production of transgenic mice (11), in part due to the silencing of the transgene of viral origin following *de novo* DNA methylation after insertion of the retroviral vector (12). Another limitation is also a relatively small size of the insert that can be carried by the vector, as well as random integration, which can influence the expression of the neighboring genes, resulting in phenotypes that are unrelated to the transgene.
2. By microinjection of DNA constructs or recently by microinjecting endonuclease-based reagents (e.g., Cas9–sgRNA–ssDNA mixture) directly into the pronucleus of fertilized mouse oocytes. First, in the case of injecting DNA constructs, the transgene is randomly integrated in a small percentage of injected oocytes as one or more tandem copies into the mouse genome, and generally all the cells of such offspring possess the transgene (13–15). The method to produce transgenic progeny is relatively quick, but it includes the risk that the DNA may insert into a critical locus that can cause an unexpected, detrimental genetic mutation. Second, the transgene may insert into a locus that is subjected to gene silencing (16). Third, if the DNA construct inserts as multiple tandem copies, it can produce extreme overexpression leading to non-physiological phenotypic effects, but more often such tandem transgene integrations are silenced in subsequent generations. In the case of using a new endonuclease approach, reagents are also microinjected into the fertilized eggs, but here the genetic modification is produced at a targeted site albeit also with some off-target events. More on this novel, CRISPR-Cas9-based method is described below.
3. The third approach is called the “gene-targeted transgene approach.” It includes the targeted manipulation of mouse embryonic stem (ES) cells at selected loci by introducing primarily loss-of-function mutations (11). Genetically modified ES cells are then microinjected into the mouse blastocysts and transferred to pseudopregnant recipient mice. The ES cells and donor blastocysts derive from mouse lines with different coat colors, and thus successful incorporation of targeted ES cells into the developing embryo of donor blastocyst results in chimeric offspring exhibiting variegated coat color. Chimeric offspring are further mated with wild-type mice to test for germline transmission of the transgene. If chimeric mice showing variegated coat color (meaning they are somatic chimeras) are also germline chimeras, then the cross with wild-type mice will result in a certain percent of heterozygous progeny carrying the transgene. Finally, the intercross of such heterozygotes produces homozygous mutant mice at an expected 25% frequency unless the mutation is detrimental to embryo survival and development (11, 17, 18). Various experiments are then carried out on mutant homozygotes to test the functionality of the genetic modification.

## Types of Transgenic Mice

The production of transgenic mice described above is time-consuming as it can take several years to establish a mutation in the ES cells and develop and validate a new transgenic mouse model. Nevertheless, traditional transgenic mice are widely used in preclinical research in oncology as well as in other research fields. One of the main drawbacks of using transgenic mice in preclinical research is the long time required to generate new transgenic mouse lines. For example, the production of transgenic mice by gene targeting (Figure 1C) requires very efficient targeting of ES cells, the generation of germline chimeras, at least two generations of crosses to obtain homozygotes, colony expansion of homozygous gene-targeted mice and only then can the characterization of oncological phenotypes be performed. If the gene-targeted mutation is dominant (i.e., heterozygotes express the oncological phenotype), the procedure is one generation shorter but still long. In all the methods described in Figure 1, the process of generating and characterizing transgenic mice takes several years, and in addition to being time- and labor-consuming, it also requires substantial financial support. In addition, one of the shortcoming of traditional transgenic mouse models (Figures 1A,B) is that a substantial fraction of mice can exhibit heterogeneity in their phenotypes, including differential tissue or cell type expression or the generation of additional phenotypes that are not related to the transgene due to the site in the genome-integration effects, whereby the transgene affects the expression of neighboring genes or epigenetically alters a larger region in *cis*. This additional variability in phenotypes results in an increased number of mice that are required to generate the transgenic mouse model that is stable (19). However, this phenomenon is not in line with the 3R principles, especially the principles “reduce” and “refine” (20). For all these reasons, new ways of generating transgenic mouse models have emerged, not only with alternative ways of modifying DNA that will be discussed later but also with the use of non-germline genetically engineered mouse models (nGEMM) that do not carry DNA modifications in germline cells but only in some somatic cells. In addition, classical transgenic mouse models can be improved by inducible or conditional transgenesis techniques.

Roughly, transgenic mice can be divided into two groups considering the loss or gain of function.

### Loss of Function

Transgenic mice, in which the gene is depleted or silenced to cause a loss of gene function, are called knockout mice. These mice provide valuable clues about the biological function of a normal gene. In translational cancer research, this represents a powerful tool in assessing the potential validity of targeted therapy because the targets can be precisely inactivated in the setting of a developing or developed tumor. In addition, studies using knockout mice are important to elucidate the cause and effect relationship in cancer development. Such studies with knockout mice can be applied for the assessment of many gene classes, including oncogenes, tumor-suppressor genes, and metabolic (“housekeeping”) genes (3). The loss-of-function models can also include dominant-negative transgenic mouse models that carry specific mutations that disrupt the activity of the wild-type gene either by overexpression or other types of modifications of gene structure. For example, dominant-negative transgenic mice have been used to probe the function of E-cadherin and its causal role in the transition from adenoma to carcinoma (21).

Two types of knockout mouse models are frequently used: constitutive (permanently inactivated target gene expression in every cell of the organism) and conditional (inducible inactivation of gene expression), which can affect a specific target tissue (tissue or cell typespecific) or can occur in a time-controlled manner (temporal) (22).

#### Constitutive Knockout

A constitutive knockout mouse generated by a procedure described in Figure 1C is often referred to as a conventional or whole-body knockout model. It defines a mouse model in which the target gene is permanently inactivated in the whole animal, in every cell of the organism (23). These mouse models can be used to assess the changes in a mouse’s phenotype, such as anatomy, physiology, behavior, and other observable characteristics. In cancer research, knockout mouse models have been invaluable for the identification and validation of novel cancer genes. For example, constitutive knockout models were used to identify the role of the newly discovered gene sushi domain containing 6 [*Susd6*, human synonym; drug-activated gene overexpressed *(DRAGO*)] as a new p53-responsive gene induced after the treatment with drugs that interfere with DNA. The results of that study showed that deletion of both *Drago* alleles in *p53−/−* or *p53+/−* mice caused statistically significantly accelerated tumor development and a shortened lifespan compared with that of *p53−/−* or *p53+/−* mice that bore wild-type *Drago* alleles (24). Nevertheless, the use of constitutive knockouts in cancer research is limited because they do not imitate the sporadic development of a tumor growing from a single cell in an otherwise normal environment (clonal evolution of tumors). Additionally, simple knockouts are frequently intended to lead to a loss of protein function (and lately in non-coding RNA genes), whereas in cancer, a subset of cancer-causing mutations consistently also results in a gain of function (25). Furthermore, one of the major drawbacks of constitutive knockouts is that germline loss of function often leads to embryonic lethality, severe developmental abnormalities or adult sterility, making conclusive determinations about tumor-suppressor genes more difficult (26).

#### Conditional Knockout

Due to the limitations of the constitutive knockout model, modification of the knockout model emerged to lead the development of conditional knockout models. The conditional knockout model can more efficiently mimic spontaneous carcinogenesis because in humans, tumors evolve in a wild-type environment, and therefore, the timing of gene loss may be a critical factor in disease development (3). Thus, to avoid and/or improve the limitations of the constitutive knockout, conditional models, in which the gene knockout can be spatially and temporally regulated, were developed. The main actors in this technology are bacterial Cre and yeast FLP enzymes, which act as site-specific recombinases to catalyze recombination between specific 34-bp loxP and FRT sites, respectively. When Cre or FLP proteins are expressed, homologous recombination is induced between loxP or FRT sites. These sites flank the gene of interest and are oriented in the same direction, which causes the deletion of the gene of interest after recombination of flanks of genetic sequence. Expression of the recombinase can be controlled temporally or spatially, and therefore, we can control the deletion of the gene of interest in temporal and spatial manners, thus overcoming interferences due to developmental abnormalities and lethality (27).

For spatial control, mice carrying the Cre or FLP recombinase under the control of a tissue-specific or inducible promotor must first be developed, usually *via* the method described in Figure 1B. When these mice are crossed with gene-targeted mice carrying the gene of interest flanked by loxP or FRT sites (developed *via* the procedure in Figure 1C), the target gene in the progeny can be conditionally inactivated in a specific tissue or cell type or at specific times during development (3). Tissue-specific knockout models can also be produced by viral driven inducible vectors delivered locally or topically by injection to infect the cells, thereby delivering Cre or FLP enzymes to target tissues or cells. This method creates regional knockout of cells within the applied area (28). Both adenovirus and lentivirus vectors can be used (29).

For the temporal control of Cre expression, tetracycline and tamoxifen-inducible systems are mainly used (30). In the case of tetracycline-based system, a transactivator and an effector are used. The tetracycline-controlled transactivator (tTA) protein binds to the tetracycline operator (tetO) that controls the activity of Cre expression to generate conditional knockouts. When adding tetracycline to the animal’s drinking water, the ingested drug binds to tTA and inhibits the association with tetO, blocking gene transcription (31). This is called a Tet-off system, wherein gene expression is inhibited in the presence of tetracycline. When using the Tet-on system, reverse tetracycline-controlled transactivator (rtTA) protein binds to tetO only if it is bound to tetracycline. Therefore, the presence of tetracycline in the animal initiates gene expression (32). One of the shortcoming of this system is the leakiness of rtTA, which compromises the desired regulation of transgene expression. The rtTA maintains some affinity for tetO sequences even in the absence of tetracycline, which results in the undesired transcription of target genes (33).

In the tamoxifen-inducible system, the Cre recombinase gene is fused to a mutated ligand-binding domain of the human estrogen receptor (Cre-ER(T)) that is specifically activated by tamoxifen. When active tamoxifen metabolite 4-hydroxytamoxifen is absent, the ER fusion protein is excluded from the nucleus. After binding to tamoxifen, the ER fusion protein is again transported to the nucleus, enabling the binding of Cre recombinase to DNA. Therefore, temporal expression of Cre can be controlled by delivering or withholding tamoxifen to the animals (34). Conditional knockout mouse model have been used in many different studies, including those manipulating genes, such as *K-Ras, Myc*, and *p53* (25), as well as in studies evaluating tumor-initiating cells. For example, the development of abnormal differentiated Schwann cells, which serve as neurofibroma tumor-initiating tumor cells, has been shown to result from the conditional loss of *Nf1* in fetal neural crest stem/progenitor cells of the Schwann cell lineage (35). One limitation of the tamoxifen-inducible system is the leakiness of the Cre-ER models, which causes a certain level of nuclear translocation of Cre-ER even in the absence of tamoxifen (36). Such an event can cause an undesired gain or loss of functional mutations (37).

Like any strategy, the production of conditional knockout models also has drawbacks and limitations: the procedure used to develop these models is lengthy and requires additional transgenic Cre or FLP transgenic models, with the possibility of mosaic expression of Cre or FLP-driven transgenes as many Cre lines are prone to both temporal and spatial ectopic expression, genetic background effects, or even eventual silencing of the expression of Cre or FLP-driven transgenes in mice in later generations (38). However, compared with the constitutive knockout, conditional knockout mutagenesis is advantageous because it uses subtler genetic modifications to examine the functional role(s) of gene(s) in a tissue or temporal manner. It also avoids potential embryonic lethality from the constitutive knockout approach, making it possible to study essential genes.

### Gain of Function

Gain-of-function studies are often used to study oncogenes in mouse models. Knock-in models of oncogene overexpression can be used to study how the oncogene drives carcinogenesis *in vivo*.

#### Constitutive Random Insertion Model

The conventional random insertion mouse model can be produced by viral vector-based transfection of early mouse embryos or by pronuclear injection of the transgene directly into fertilized oocytes (Figures 1A,B). The transgene is then randomly incorporated into the genome. Although the procedure is very straightforward and relatively simple, the random incorporation into the genome is the main drawback of this model because it can result in an undesirable expression level or spatiotemporal distribution of transgene activity, or even deleterious effects, thus limiting the usefulness of the model. These models have been widely used to study how oncogenes such as *K-ras* drive tumorigenesis *in vivo* (39–42).

#### Knock-in Permissive Locus Model

To overcome the limitations of the constitutive random insertion model, several new models have been developed to study the gain of function, specifically by inserting a gene of interest into a specific region of the genome. Using homologous recombination, a more predictable and stable gain-of-function model can be obtained. The most commonly used site is the *Rosa26* locus because it does not contain any essential genes and provides stable and predictable expression of the transgene in various cell types (43, 44). *Npm1* transgenic mice can serve as a good example of a mouse model using the *Rosa26* locus and Cre-regulated expression. The *Npm1* mutation, which is the most frequent genetic alteration in acute myeloid leukemia (AML) (45), can be characterized in this knock-in permissive model. With this model, it has been shown that *Npm1* mutations affects megakaryocytic development and mimics some features of human *NPM1*-mutated AML, thus serving as a good model for further investigations of AML (46).

#### Conditional Knock-In Model

As previously pointed out, the constitutive gene knock-in described in Section “Constitutive Random Insertion Model” can lead to lethality, sterility, and developmental defects. Therefore, similar to the knockout mouse models, spatial and temporal control of the gene has to be regulated to also circumvent these limitations in knock-in models. Conditional knock-in models can be generated using tissue-specific promotors or by inserting a strong translational and transcriptional termination (STOP) sequence flanked by loxP or FRT sites between the promotor sequence and the gene of interest (47). When the STOP sequence is present, transcription of the gene interest is blocked. However, when Cre or FLP recombinase are expressed and present, the STOP cassette is removed, allowing expression of the gene (28). Thus, gene expression is mediated by excision of the STOP cassette and recombinase expression. Therefore, gene expression is spatially, temporally, and inducibly mediated by Cre or FLP systems (48). Occasionally, also depending on the knock-in genome site, the STOP cassettes can be leaky, as observed in the initial *K-ras* G12D models of lung carcinoma, wherein death due to respiratory failure prior to tumor progression occurred (49). Improved STOP cassettes with less leakiness were subsequently developed. Later versions of conditional knock-in mouse model of *LSL-K-ras* G12D were shown to be good models to study the initiation and early stage pulmonary adenocarcinoma, allowing control of the timing, location, and number of tumors (28). Additionally, conditional knock-in mouse models were also used to investigate the role of *Brca1* RING function in tumor suppression and therapeutic response, where it was determined that *Brca1* RING did not affect resistance to therapy (50).

#### Reporter Knock-In Model

To observe the expression of the targeted gene at the transcriptional or translational level, reporter knock-in mouse models can be used. Genes encoding fluorescence proteins have been widely used as reporters in biomedical research and frequently employed to analyze the transgene activity. In reporter models, transgenes are used for the visualization of proteomic, metabolic, cellular, or genetic events *in vivo*. The most commonly used technique for visualization is fluorescent and bioluminescent optical imaging due to the increased sensitivity, relative inexpensiveness, and less time-consuming and more user-friendly features compared with those of, for example, histological, genetic, or biochemical methods. Furthermore, such models are also in line with the 3R principles in research using animals, incorporating at least two of these principles; reduction (less animals used) and refinement (less harmful methods) (20). Several transgenic mouse lines are available that express reporter genes (51). The most common reporters are green fluorescent protein (GFP) and red fluorescent protein (RFP) (52, 53) for fluorescence and firefly luciferase for bioluminescence (54). The latter can also be used for the visualization of tumor growth *in vivo*. To study tumor cell proliferation *in vivo*, mice expressing firefly luciferase under control of the human *E2F1* gene promotor, which is active during proliferation, were crossed with a mouse cancer model (55).

Recently, in cancer research, detection and imaging of the immune response has become one of the fundamental ways to follow the treatment course in live animals. Because several new treatments aim to modulate the immune response, the recruitment of immune cells to tumors is an important indicator of the effectiveness of anticancer immune therapies. This recruitment can also be used to observe tumor-induced immune suppression. The interactions between cells of the immune system and tumor targets in the context of the tumor microenvironment can be followed by intravital microscopy (56). One of the immune cells that is closely connected to progression of the various types of cancers is the regulatory T cell, the action of which is based on the immunosuppressive function of the immune response (57). For example, Bauer and colleagues used two-photon microscopy to investigate the pro-tumor role of tumor-experienced regulatory T cell interactions with dendritic cells. Transgenic mice expressing enhanced GFP (eGFP) in all T cells and mCherry in antigen-presenting cells were used. Their study showed that regulatory T cell interactions with dendritic cells in tumor-draining lymph nodes caused the death of dendritic cells (58). Intravital microscopy is a valuable tool also for assessing the dynamic changes in the tumor vasculature and following the transcriptional targeting of gene expression in various tissues (59, 60).

## New Mouse Models for Cancer Research

New mouse models have emerged for research in preclinical oncology, especially within the last decade with the great advancements in molecular biology as well as genomic technology and engineering. One novelty was the production of nGEMM, which showed great promise in producing transgenic mice at a low cost with less time-consuming procedures. Other novelties are alternative DNA modification techniques, which are more efficient for the faster and cheaper generation of new transgenic mice. All the different alternative modifications of DNA can be used to produce new types of transgenic mice or further modify conventional models, as described in Figure 1. Furthermore, apart from transgenic mice, new models used in cancer research have emerged, such as humanized mice, which have re-established borders in tumor microenvironment studies. Humanized mice implemented with patient-derived xenografts (PDX) can elucidate the interaction between the human tumor and human immune system as part of the tumor microenvironment in a mouse model.

### Non-Germline Genetically Engineered Mouse Models

Non-germline genetically engineered mouse models are characterized as mouse models carrying genetically engineered alleles in somatic cells but not in germline cells (22, 61). A comprehensive review of their advantages and limitations is provided elsewhere (19), and only a brief summary is given herein. The nGEMM are produced by two major approaches: by generating chimeric or transplantation models. Non-germline chimeric mice can be a by-product of traditional knockout technology (Figure 1C), presenting chimeric mice that do not carry modified ES cells in the germline lineage (62). Chimeric mouse models for cancer research can also be produced only for the purpose of generating nGEMMs by injecting genetically engineered, cancer predisposed, ES cells into blastocysts from a chosen genetic background to develop cancer-prone chimeric mice in somatic tissues (63). As recipient blastocysts usually have a wild-type genetic background, and not every cell in the body is hence genetically modified, this situation better models carcinogenesis in humans. Large banks of genetically modified mouse ES cells in a large proportion of genes have already been established in genome-wide mutagenesis programs such as European Conditional Knockout Mouse Mutagenesis, North American Conditional Knockout Mouse Mutagenesis, the USA-NIH Knockout Mouse Project, and the European Mouse Disease Clinic projects, and they are also commercially available (e.g., https://www.jax.org). Hence, ES cells obtained from these resource centers can be immediately used to generate tailored nGEMMs. As carcinogenesis is spatially and temporally restricted, tissues or developmental time specificity can be accomplished by applying induction reagents locally or in a time-restricted manner. One limitation of chimeric nGEMMs is the increased variability related to tumorigenesis between individual chimeric mice and that ES cells can populate different cell lineages and hence different target organs, which can produce heterogeneity between individual mice (49, 64). Additionally, some of the target cell lineages cannot be efficiently or cannot at all be populated by ES cells.

In transplantation models, the transplanted tissue can derive either from genetically engineered donor mice that can have a predisposing cancer mutation or from mouse or human cells that have been previously engineered *ex vivo* (mouse-to-mouse; human-to-mouse models of nGEMM) (19). Transplantation systems have first and mostly commonly been adapted to study hematopoietic carcinogenesis. Here, hematopoietic stem and progenitor cells are derived from bone marrow or fetal liver and transplanted by simple intravenous injection into lethally irradiated recipient mice (65). Irradiation models of high-dose chemotherapy in humans also create a window for successful engraftment of transplanted modified hematopoietic stem and progenitor cells into nGEMMs (66, 67). Taken together, nGEMMs are very potent for their use in cancer research, with great value in testing new therapeutics. One of the greatest advantages compared to traditional GEMM is the faster generation of new transgenic mice and improvements in modeling the tumor microenvironment, which is more similar to the situation in human carcinogenesis.

### Alternative DNA Modification Techniques

Fine-tune modeling of mouse cancer models can be performed using several alternative methods that have been developed for faster and more reliable testing of genes for their oncogenic potential. The most commonly used methods are transposon-based insertional mutagenesis, RNA interference (RNAi), and engineered nucleases (68).

#### Transposon-Based Insertional Mutagenesis

**Figure 2:**
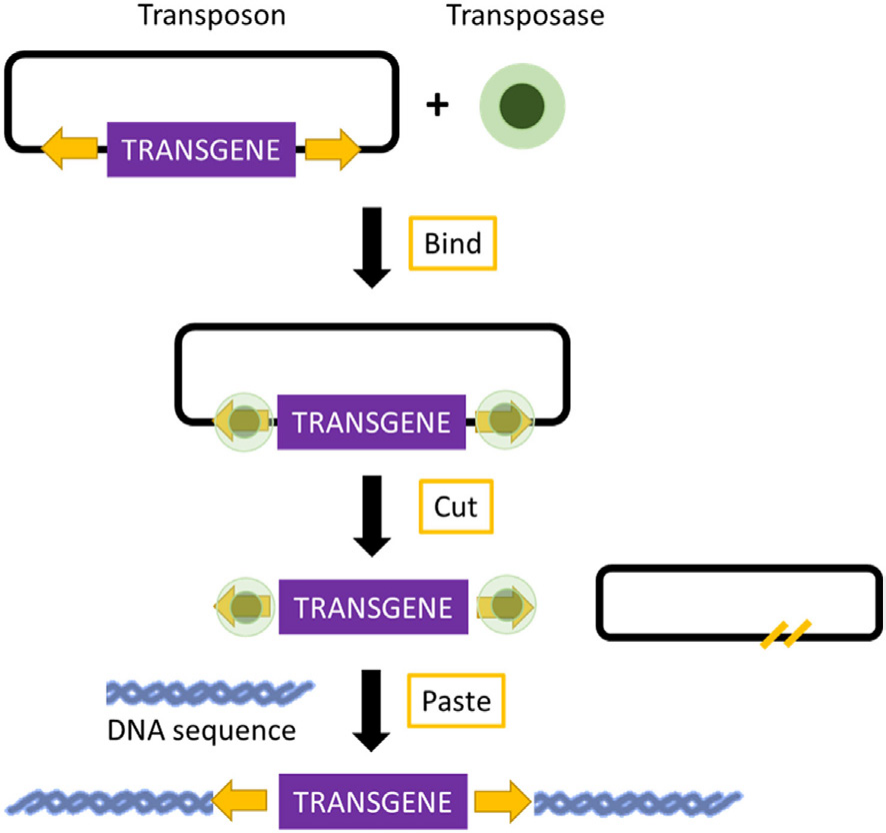
Mechanism of transposon-based insertional mutagenesis. The transposon system is composed of a transposon (targeted sequence of DNA) and an enzyme (transposase). The transposase binds to the appropriate site along the transposon and excises the transposon. It then pastes the transposon at an appropriate location in another DNA sequence, TA site in the case of Sleeping Beauty, and a TTAA site in the case of PiggyBac.

Transposons are DNA sequences with the ability to move from one location of the genome to another. Two groups of transposons are known: retrotransposons and DNA transposons. Retrotransposons, because of their low integration efficiency, the integration of incomplete retrotranscribed elements, and the concomitant induction of chromosomal aberrations, are rarely used for the production of transgenic mice (69). In contrast, DNA transposons have shown great promise in transposon-mediated insertional mutagenesis. Mutagenesis relies on a transposase enzyme, which distinguishes specific DNA sequences and “cuts” the DNA between them. The excised DNA is then reintegrated at another site in the genome (70) (Figure 2). Transposon-based insertional mutagenesis can hence be used in genetic screens to identify novel cancer-causing genes, such as oncogenes or tumor-suppressor genes (71). The two most effective transposons are described: Sleeping Beauty and PiggyBac.

##### Sleeping Beauty

The important elements of Sleeping Beauty are the transposase, which is an enzyme used for the mobilization of DNA, and the transposon, which is a mobilized sequence of DNA (72). The mechanism of Sleeping Beauty relies on a cut-and-paste mode, and when the transposase excises a transposon, it leaves behind a three-base footprint. Then, the transposon can mobilize to any location in the genome where a TA dinucleotide is present. There are more than 300 million TA sites in the genome. The TA insertion site is duplicated during the process of transposon integration (70, 73). The transposon can carry any sequence of choice, but the transposition efficiency decreases with an increased size of the sequence (70). These sequences can be mutagenic elements, which can be intended to imitate those present in retroviruses. The Sleeping Beauty transposons can be used for the induction of loss-of-function mutations as well as gain-of-function mutations (74). Due to the cut-and-paste mode, there is only a 40–50% possibility that reintegration of the excised transposon will occur into the genome. Additionally, because the number of transposons integrated in the genome decreases over time, a large number of transposable elements are required (75). Sleeping Beauty can be used for the discovery of candidate cancer genes and to search for the drivers of multiple cancer types. Because of these screens, several cancer-promoting mutations candidate have already been found, which can be used in the development of new mouse models that may prove useful for therapeutic testing. To identify candidate drivers of colorectal carcinoma, transposon-based screens are useful because cancer-promoting mutations are caused by transposon insertion events rather than genome-wide instability. Colorectal carcinoma has been modeled using mice carrying the mutagenic *T2/Onc2 SB* transposons, conditional *Rosa26-lsl-SB11* transposase, and *villin-Cre* to activate transposition specifically in gastrointestinal tract epithelial cells. Using this technique, intraepithelial tumors, adenomas, and adenocarcinomas in the small and large intestines were generated. Moreover, analyses of the transposon insertion site of these tumors identified 77 candidate colorectal carcinoma genes, 60 of which are known to be altered or dysregulated in human colorectal carcinoma (76). Furthermore, Sleeping Beauty can also be used to induce cancer in a tissue of interest by combining it with the Cre recombinase inducible system. By employing Cre expression under the control of an albumin enhancer or promotor sequence, which is specific for liver, Sleeping Beauty transposition was limited to the liver. This system was used to screen for hepatocellular carcinoma associated genes. New genes potentially involved in carcinogenesis, such as UBE2H, were discovered, and therefore, this modified system was introduced in the search for new possible candidate genes (77).

##### PiggyBac

PiggyBac transposons are the only efficient alternative to Sleeping Beauty for cancer gene discovery. Compared with Sleeping Beauty, PiggyBac can carry larger cargos (up to several hundred kilobases). These cargos are inserted with higher transposition activity into mammalian genomes. Additionally, the PiggyBac system requires a TTAA insertion site instead of TA, and after the transposase excises a transposon, it does not leave any footprint, in contrast to Sleeping Beauty. This imprecise excision of PiggyBac can lead to damage at the mobilization site, thereby creating loss or gain-of-function alleles (70, 78). The PiggyBac transposons can also be used to generate transgenic rodents expressing a reporter fluorescent protein in different organs. Recently, transgenic rats carrying either the RFP gene or the eGFP gene were generated by injecting pronuclei with PiggyBac plasmids. Not only did the transgenic rats express the RFP and eGFP gene in many organs, but they also had the capability to transmit the reporter gene to the next generation through integration into the germline lineage (79).

#### RNA Interference

RNA interference in mice represents an alternative to knockout mice, or, more accurately, a knockdown mouse. Namely, knockdown by RNAi does not generate a completely loss-of-function allele (80). Silencing, or better, downregulating gene expression of a target gene by small interfering RNA (siRNA) has been mainly used to study gene function (81). It was used for silencing estrogen receptor alpha (*ESR1*), where stable knockdown suppressed the proliferation and enhanced apoptosis of breast cancer cells (82), or for silencing transketolase (*TKT*), which affects cell proliferation and migration as well as interactions with other metabolism-associated genes in lung cancer cells (83). However, the knockdown effect of siRNA is only transient due to the short half-life of siRNA molecules. To achieve a more sustained gene-silencing effect, plasmids encoding short hairpin RNAs (shRNA) can be used. RNAi by shRNAs permits reversible silencing of gene expression without altering the genome. To increase the expression of the shRNA, the targeting vector of interest can be inserted into the *Rosa26* locus by the recombination of a site-specific recombinase in ES cells (developed using a technique described in Figure 1B).

#### Engineered Nucleases

Thus far, three kinds of engineered nucleases have been developed and tested for DNA modulation: zinc-finger nuclease (ZNF), transcription activator-like effector (TALEN) nuclease, and the latest clustered regularly interspaced short palindromic repeat (CRISPR)/-associated (Cas9) system (84).

Briefly, ZNFs and TALENs are produced by combining a DNA-binding domain with a DNA-cleavage domain. These domains can be engineered to act as a site-specific nuclease, cutting DNA at strictly defined sites, which enables zinc-finger or TALEN nucleases to target unique sequences within complex genomes. The targeting efficiency of the ZNF system reaches 68% (85), and ZNF-mediated gene-targeting experiments are a relatively efficient means for generating non-homologous end-joining (NHEJ)-mediated knockout mice (86). Using the TALEN method to produce knockout mice is efficient in 49–77% of cases (87), which can be increased with a greater concentration of TALEN mRNA. This method has been primarily used to increase the efficiency of gene targeting, and compared to ZNFs, TALENs yield higher mutation efficiencies and survival rates.

However, the use of ZNFs and TALENs is limited because construction of the protein domains for each particular genome locus is complex and expensive. Additionally, single nucleotide substitutions or inappropriate interaction between domains can cause inaccurate cleavage of the target DNA (84). Furthermore, the targeting efficiency may be variable and much lower than reported above. However, a major drawback is that simultaneous gene targeting in multiple genes is hindered, preventing studies of oncological phenotypes wherein multiple mutations are required, in analyses of gene family members with redundant functions or in cases of cancers in which gene–gene interactions exist.

##### CRISPR/Cas9 System

**Figure 3:**
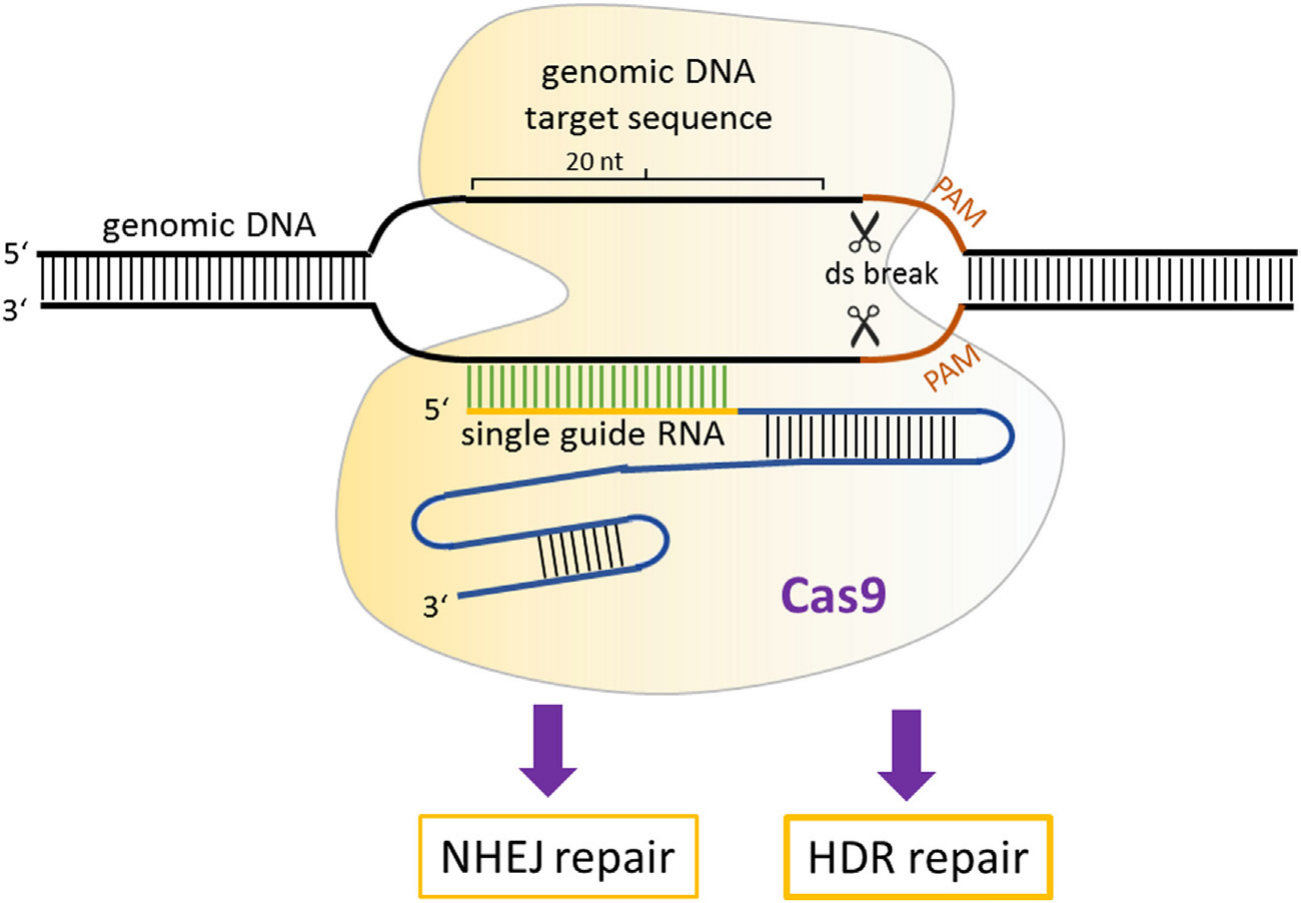
Mechanism of CRISPR/Cas9 gene modulation. A single guide RNA directs Cas9 nuclease to a genomic locus, where it cuts the target sequence in the presence of protospacer-adjacent motif. The resulting double-stranded breaks stimulates DNA repair, which can occur *via* non-homologous end-joining or homology-directed repair-mediated repair.

The simplest and the most effective engineered nuclease system to generate transgenic mice is the CRISPR/Cas9 system (88). Compared with ZFNs and TALENs, the CRISPR/Cas9-mediated genome editing is more efficient, and the design, construction of reagents, as well as delivery are easier. Additionally, targeted mutations in multiple genes (so-called multiplex genome engineering) are possible with the CRISPR/Cas9 system. This system consists of a Cas9 nuclease, which can be directed to any genomic locus by an appropriate single guide RNA (sgRNA). Until now, three main types of Cas9 variants have been developed that differ in their mechanisms of action. The first system to be adapted for mouse transgenesis was the wild-type Cas9 protein from the type II CRISPR system of *Streptococcus pyogenes*, which functions *via* an association with the sgRNA with a relatively short recognition sequence (~20 nt) (89). For double-strand cleavage, this system requires the protospacer-adjacent motif (PAM), which is “NGG” or “NAG” for *S. pyogenes* Cas9 at the 3′ end of the target sequence. Recently, new forms of Cas9 enzymes have also been developed that can bind to alternative PAM sites and thereby extend the range of utility of Cas9 (90). Once the double-stranded breaks occur, it can be repaired by NHEJ or by homology-directed repair (HDR) (91) (Figure 3). NHEJ-mediated repair frequently results in short insertions or deletions that generate loss-of-function mutations.

The second Cas9 variant was developed (92) to increase the efficiency of HDR, allowing insertions or replacements of specific nucleotides. A mutant form Cas9 protein (Cas9D10A, called nickase) was developed that cleaves only one DNA strand, downregulating the activation of NHEJ. When a homologous repair DNA template with a specific mutation or sequence to be introduced is provided in the mixture of the sgRNA and Cas9D10A mutation, it can serve as a template to repair the lesion. This activates the high-fidelity HDR pathway and hence offers the possibility to generate allele replacements and other specific modifications in the mouse genome that were essentially impossible with the classic transgenesis methods described in Figure 1.

The third Cas9 variant is the so-called “dead” Cas9 or nuclease-deficient Cas9 (dCas9) (93), in which certain mutations were introduced to inactivate the cleavage activity but retain the DNA-binding activity. This variant was developed to be able to target any region of the genome without cleavage and by fusing dCas9 with various activator or repressor domains, to up- or downregulate the transcription of target genes. An additional application of the dCas9 system was developed by Chen and Huang (94). By fusing dCas9 to eGFP, they developed a visualization tool and demonstrated that they could visualize several dynamic processes, such as telomere dynamics during elongation or disruption, subnuclear localization of certain loci, and dynamic behavior during mitosis in living human cells.

##### Application of the CRISPR/Cas9 System in Oncology

Several successful applications of the CRISPR/Cas9 system in cancer research have been published by using one of the aforementioned three systems or by combining the classic transgenic models described in Figure 1 with CRISPR/Cas9 to generate germline or nGEMM mouse cancer models (88). Some early successful attempts to develop new *in vivo* cancer models include a new pancreatic cancer model combining viral vector delivery and CRISPR/Cas9-mediated somatic genome editing (95), and a lung cancer knock-in model (96). The latter was developed by combining a Cre-dependent Cas9 mouse model with sgRNA delivery, which generated loss-of-function mutations in *p53* and *Lkb1*, as well as nucleotide replacement leading to an oncogenic *K-rasG12D* mutation that causes lung adenocarcinoma. A conditional liver-specific mutation in cancer genes was developed by Xue et al. (97), whereas the development of novel brain tumor mouse models was reported by Zuckermann et al. (98). An important step forward in new models in cancer research was demonstrated by Maddalo et al. (99), who used CRISPR/Cas9-mediated *in vivo* somatic genome editing to engineer chromosomal rearrangements. This class of mutations plays an important role in carcinogenesis, but it is very difficult, if not impossible, to develop using classical transgenesis approaches (Figure 1). Authors have used viral-mediated delivery of the *Eml4–Alk* fusion gene by the CRISPR/Cas9 system to somatic cells of adult animals, which models an inversion on chromosome 2: inv(2)(p21p23) that occurs in humans. Expression of the *Eml4–Alk* fusion gene in this model results in pathological and molecular characteristics of typical ALK + human non-small cell lung cancers (NSCLC). Moreover, this mouse model responds positively to ALK inhibitors. Similarly, using a somatic CRISPR/Cas9 approach, Cook et al. (100) demonstrated in an *ex vivo* and *in vivo* study that a chromosomal rearrangement resulting in *Bcan–Ntrk1* fusion creates a potent driver for glioblastoma development.

The adaptability of the CRISPR/Cas9 system to the scientific question and a relatively easy way to scale up the experimental design has already led to high-throughput *in vivo* screens to catalog functional tumor suppressors. One such comprehensive study by Wang et al. (101) mapped functional cancer genome variants of tumor suppressors in the mouse liver of the wild-type, immunocompetent strain. By injecting AAV pools containing a large (278) sgRNA library directed toward known and the most frequently mutated tumor-suppressor genes into Rosa-Cas9-eGFP knock-in mice, they were able to generate a mutational atlas of liver tumors. All the mice that received this AAV-sgRNA of tumor-suppressor sgRNA developed liver cancer and died within 4 months, demonstrating the validity and extremely high efficiency of this screening approach. Therefore, AAV-mediated CRISPR-Cas9 screens provide a powerful high-throughput tool for mapping functional cancer tumor suppressors in various tissues in fully immunocompetent mice.

Studies using wild-type Cas9 or nickase mutation Cas9 variant have thus far been most frequently used in mouse cancer model development. However, application of the dCas9 system in which no genome modifications are produced but an effect on the expression of target genes is observed have also started to emerge in *in vivo* models of cancer. A good example of this type of research has been described in Braun et al. (102), who aimed to examine the effect of the upregulation of *Mgmt* using dCas9 protein fused to a fourfold repeat of the VP16 transcriptional activator (VP64) in combination with sgRNAs targeting upstream regulatory regions (103). This target gene was chosen because it is known to detoxify DNA lesions caused by the chemotherapeutic agent temozolomide. Murine acute B-cell lymphoblastic leukemia cells were first infected with a combination of dCas9-VP64 and sgRNAs and transplanted into wild-type fully immunocompetent C57BL6/J mice. Positive results were obtained, as upregulation of *Mgmt* was achieved, and the mice responded to temozolomide. These findings demonstrated that the dCas9-based system could be successfully used to affect gene expression only and to model oncological genetic modifications during treatment relapse *in vivo*.

Furthermore, the simultaneous injection of Cas9 mRNA and sgRNA into the cytoplasm of zygotes has been shown to efficiently and reliably generate knockout mice with the highest targeting efficiency (67–100%) of all engineered nucleases (84). Beyond the development of novel transgenic mice, CRISPR/Cas9 can also be used to refine existing models of cancer by reengineering ES cell lines from well-known transgenic mice to harbor additional constitutive or conditional mutant alleles of oncogenes and tumor-suppressor genes (104). Therefore, CRISPR/Cas9 represents an efficient method for generating transgenic mice due to its simplicity, cost-effectiveness, high efficiency, and low fetal toxicity even at relatively high doses of Cas9 mRNA and sgRNA (105).

#### Humanized Mouse Xenograft Models

Patient-derived xenograft (PDX) models have been extensively used in studies of various solid and hematologic malignancies, such as breast cancer, colorectal cancer, pancreatic cancer, chronic lymphocytic leukemia, and large B cell lymphoma (106, 107). PDX models are used for the assessment of human tumor biology, identification of therapeutic targets, and are an important model for preclinical testing of new drugs for various cancers. PDX models are established by the implantation of cancer cells or tissues from patient primary tumors into immunodeficient mice. Several types of standard immunodeficient mice exist, such as athymic nude, SCID, NOD-SCID and recombination-activating gene 2 *(Rag2)* knockout mice (108). However, these mouse models are usually used to establish a xenograft cancer cell line or to grow transplantable tumor xenografts, and they are unable to grow primary cancer cells or tissues. To accomplish this goal, greater immunodeficiency is required, which is provided by the generation of NOD/SCID mice with *IL2rg* mutations (NSG) that are able to engraft almost all types of cancer due to their enhanced immunodeficiency (109). To implant patient-derived tumors into immunodeficient mice, small fragments of tumors, cell suspensions derived from blood or from the digestion of tumors into single-cell suspensions are used. The implantation can be performed heterotopically or orthotopically. Heterotopic implantation, for example, subcutaneously, has advantages over orthotopic implantation due to the simplicity of the method and more convenient measurement of tumor size. Subcutaneous and intravenous PDX models are most widely used in cancer research for solid tumors and leukemias. In contrast, if the main aim of the research is metastases of certain cancer types, than orthotopic models are superior because orthotopic implantation into host tissues can produce metastases via the normal process of cancer progression (110).

**Figure 4:**
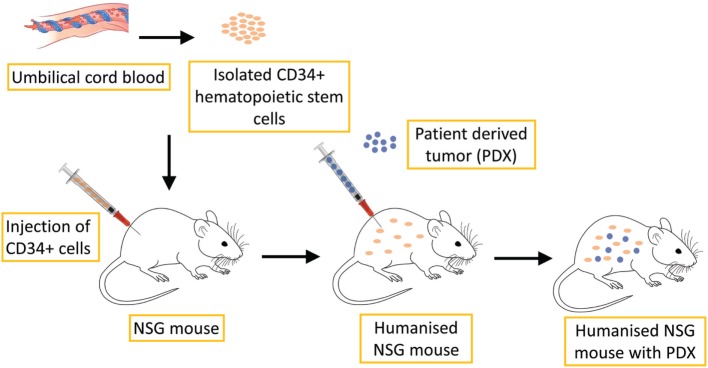
Schematic illustration of humanized PDX mouse model production. CD34+ human hematopoietic stem cells, which are isolated from umbilical cord blood, are transplanted into NSG mice. This process leads to the development of human hematopoietic and immune systems. PDX of various tumors can then be implanted for further research.

Due to recent advances in immunotherapy illuminating the importance of the immune response in tumor progression and treatment, new PDX models are necessary, namely PDX models together with the human immune system, in which the interaction between human cancers and the human immune system can be investigated, as well as potential antitumor immunotherapies (107). Several methods can be used to produce these so-called humanized mouse models. One such model can be produced by the transplantation of total peripheral blood or tumor-infiltrating lymphocytes into immunodeficient mice. However, these methods are very limited in cancer research because they cause severe graft-versus-host disease (111). Therefore, another method has been used to produce humanized mouse models through the transplantation of CD34+ human hematopoietic stem cells (HSCs) or precursor cells isolated from umbilical cord blood, bone marrow and peripheral blood, as shown in Figure 4. Transplantation of HSCs gives rise to various lineages of human blood cells in mice (112).

These humanized models can be used to investigate the efficacy and mechanism of cancer immunotherapy, such as programmed cell death protein 1 (PD-1)-targeted immunotherapy. Wang et al. (113) described the development of humanized NSG (huNSG) mice by transplantation of human (h)CD34+ hematopoietic progenitor and stem cells, which led to the development of human hematopoietic and immune systems. Subsequently, they implanted the PDX of NSCLC, sarcoma, bladder cancer, and triple-negative breast cancer into such humanized mice. They discovered that tumor growth curves were similar in huNSG in comparison to non-human immune cell-engrafted NSG mice. Treatment with the checkpoint inhibitor pembrolizumab, an antibody that targets PD-1, caused significant growth inhibition of PDX tumors in huNSG, but not NSG mice. These results suggest that tumor-bearing huNSG mice could represent an important new model for preclinical immunotherapy research. A similar result was obtained by Pan et al, (114), who investigated the antitumor effectiveness of pembrolizumab in human bladder cancer PDX in huNSG mice. They observed that treatment with pembrolizumab inhibited tumor growth and decreased the numbers of CD4+ PD1+ and CD8+ PD1+ cells in peripheral blood and increased the numbers of CD45+ and CD8+ cells in PDXs. One limitation of NSG mice is that despite engraftment with human CD34+ cells, these mice will acquire only partially fully mature human blood cells due to incompatibility between the mouse and human cytokines necessary for blood cell development. Recent models aim to achieve the combination of transgenic or knock-in mouse models expressing human cytokines together with NSG and CD34+ cell transplantation to improve engraftment (115).

Furthermore, recent studies have demonstrated that the microbial ecosystem has a major impact on the local and distant immune response and that the efficacy of immune therapies with checkpoint inhibitors, such as pembrolizumab, can be diminished by the use of antibiotics and enhanced in the presence of specific gut microbes. To fully evaluate the interplay between immunotherapies and the microbiota, new mouse models are emerging, such as specific pathogen-free mice with defined commensal bacteria or preconditioned with antibiotics, or germ-free mice lacking commensal bacteria (116). Commensal bacteria such as *Bifidobacteria* spp. and *Akkermansia muciniphila* can increase the efficiency of anti-programmed cell death protein 1 ligand (PD-L1)-based immunotherapy against epithelial tumors by improving tumor control (117–119). Additionally, a correlation between the use of another immune checkpoint inhibitor, ipilimumab (anti CTLA-4 antibody), and colonization by Bacteroidales was observed. The efficacy of CTLA-4 blockade was improved by the microbiota composition of Bacteroidales, which affects interleukin 12-dependent Th1 immune responses, thus enabling better tumor control in mice while sparing intestinal integrity (120). One limitation of these mouse models with engrafted human microbiota is that these mice are likely unable to support colonization by all commensals of the human GI tract; therefore, it may be sufficient to focus on bacteria that successfully colonize both humans and mice.

## Current Directions in Transgenic Mouse Cancer Models

The mouse cancer models discussed in the previous sections clearly show a great impact of these models on the study of basic mechanisms of carcinogenesis, as well as the evaluation or development of therapies that are potentially applicable in human oncology. However, both traditional transgenic models and new opportunities offered by CRISPR/Cas9 provide great promise in even more efficient and translatable mouse models for cancer research in the future. In this section, we discuss selected fields in which we predict major developments in the near future: personalizing humanized mice, replicating specific human mutations in mouse models, analyzing and manipulating the “cancer” epigenome, and prospects in the use of mouse models for gene therapy applications in humans.

### Personalizing Humanized Mice

Humanized mice have shown great potential in preclinical oncology studies. To further increase the potential of these models, there is a necessity for the immune system in humanized mice to be compatible with both its host environment and with the implanted tumor tissue to accurately model the patient’s immune response during treatment. Tissue incompatibility of humanized mice that are engrafted with an immune system from one person and implanted with the tumor of another could be the reason for the immune response observed in humanized mice, which is thus not related to the specific treatment applied to the mice. When humanized mice are produced from the engraftment of CD34+ cells, some of the mature xenoreactive T cells are also introduced into these mice. These T cells differentiate within the engrafted bone marrow, mature within the mouse and seem to display some xenoreactive tendencies (108). However, because the transplanted human immune system is weakened, it prevents complete rejection of the xenograft. One possible solution to this problem could be the production of a humanized xenograft model in which the CD34+ cells and implemented tumor tissue are derived from the same donor. Klein et al. produced humanized mice using CD34+ blood cells isolated from biopsied bone marrow of breast, lung, prostate, or esophageal cancer patient, raising the possibility of individualized analyses of antitumor T cell responses (121). Moreover, a new melanoma PDX model has been designed wherein tumor cells and tumor-infiltrating T cells from the same patient are transplanted sequentially in NOG/NSG knockout mice. This model was developed to study the most advanced and most promising current anticancer therapies, immune checkpoint inhibitors and adoptive cell transfer of autologous tumor-infiltrating T cells that have demonstrated complete durable responses in a subpopulation of patients with advanced melanoma (122).

### Replicating Specific Human Cancer Mutations in Mouse Models

The conventional mouse models described in Figure 1 will continue to be used in cancer research both on their own and in combination with other approaches such as transplantation models and humanized mice. However, as alluded previously, all three major traditional transgenesis techniques suffer due to an inability to efficiently develop precise allele replacements or insertions. Initially, CRISPR/Cas9-mediated mutagenesis was highly effective for generating loss-of-function models but not precise allele replacements or gain-of-function mutations, which are most frequent in cancer. However, recent improvements in the CRISPR/Cas9 system have immensely increased the efficiency of HDR [*e.g*., Gutschner et al. (123); Komor et al. (124)] and hence the ability to engineer precise mutations at any site in the genome. Some successful allele replacements in the cancer research field have also been achieved *in vitro*. For example, Burgess et al. (125) developed the homozygous replacement of the oncogenic *G13D K-RAS* mutation in a human colorectal cancer cell line, which rendered them sensitive to drug treatment. One major novelty of the CRISPR/Cas9 system is the ability to simultaneously generate multiple mutations. One such successful attempt was relayed in a study by Walton et al., who managed to generate triple gene mutations that made cells deficient in *Trp53, Brca2*, and *Pten* genes (126). Novel gene fusion mutations are frequently found in human cancers. To model one such fusion in mice linking *Dnajb1–Prkaca* genes into one transcript, Engelholm et al. (127) employed CRISPR/Cas9 method to precisely delete a region in mice that is syngeneic to the human region on chromosome 8 to recreate a *Dnajb1–Prkaca* fusion. They demonstrated that this fusion is the only driver to induce hepatocellular carcinoma, with several features resembling human liver cancer.

Apart from precise mutations encompassing one nucleotide or smaller genomic segments, as described above, CRISPR/Cas9 technology also offers opportunities to generate large chromosomal aberrations. Recently, studies have been published with the aim to improve the efficiency of generating chromosomal rearrangements. One such strategy, named CRISpr MEdiated REarrangement strategy (128), has proven very efficient in producing desired rearrangements from one single experiment. Targeted large deletions (up to 24.4 Mb), duplications, and inversions in rodent models were developed using this approach, which will probably soon be used in cancer research to model chromosomal aberrations involved in tumor biology.

Cancer is also characterized by multiple epigenetic changes that can drive carcinogenesis and confer resistance to treatment. Epigenome editing, especially by the CRISPR/Cas9 system, now allows analyses of precise epigenetic modifications and their effects on cancer development and therapy. One great challenge ahead will be to achieve the reversion of epigenetic modifications, including DNA methylation and other mechanisms (e.g., histone acetylation) at precise sites and ensure that such an intervention is mitotically heritable. Some recent studies in cell lines have demonstrated that selective epigenetic changes (e.g., DNA methylation) can be achieved with the expected outcome on the expression of target genes (129). Apart from DNA methylation, posttranslational modifications of proteins, such as histone acetylation, also present an important epigenetic mechanism of gene expression disruption that can lead to carcinogenesis. In a recent study by Shrimp et al. (130), dCas9 fused to an activator, p300, to control the expression of lysine acetyltransferases (KATs) was applied. This pioneering study demonstrated the potential of the dCas9-p300 system for studying gene expression mechanisms in which acetylation plays a causal role, which is certainly the case in cancer biology. Further developments in this area of research may lead to the development of methods for the spatiotemporal control of acetylation at specific loci, which in turn could lead to therapeutic effects. The ability of the dCas9-effector system to activate or repress endogenous gene expression also provides a new and unique opportunity to further examine cancer-associated *cis* or *trans* acting regulatory non-coding RNAs. Thus, recent developments in CRISPR/Cas9 technology demonstrate great promise for future use and application in transgenic mouse models for studying cancer biology.

### Delivery Methods

In transgenic mouse models, the delivery of components to induce mutations or to deliver modified cells *in vivo* still presents a major challenge. A brief review of the delivery methods used in cancer mouse models is provided below, with a focus on the CRISPR/Cas9 system. The development of delivery vehicles for CRISPR/Cas9-mediated transgenesis, especially in the generation of *in vivo* mouse cancer models, has been challenging because of the requirements for the delivery of multiple components in a spatially or temporally controlled manner. Nevertheless, some delivery methods have already been attempted in mouse models of cancer and vary widely depending on the target cancer type or scientific questions asked.

Intravenous injection of Cas9-edited hematopoietic stem progenitor cells has been successfully applied to model myeloid malignancies in mice (131) and in a Burkitt lymphoma model (132). Electroporation-based delivery, a widely used method for the introduction of different molecules (chemotherapeutic drugs and genetic material) into different types of cells *in vitro* and *in vivo* (133), has also been used in *in vitro* cancer modeling, for example, in modeling alveolar rhabdomyosarcoma in mouse myoblasts (134) as well as *in vivo* for hematopoietic cell-based therapy of malignancies (135). A so-called hydrodynamic tail vein injection of CRISPR/Cas9 components has been applied in a high-throughput multiplex-mutagenesis liver cancer screen (136). Similarly, in a genome-wide screen of lung cancer in mice, subcutaneous injections were used (137). For NSCLC, basic epithelial cell transfection has also been used to target genomic rearrangements (138). To develop transgenic mouse models harboring CRISPR/Cas9-induced mutations in every cell of the body, classical microinjections into fertilized eggs or blastocysts (for modified ES cells) are frequently employed (139). Recently, some successful attempts utilizing the electroporation of pronuclear zygotes have also been reported (140, 141). Transfection with the polyethyleneimine reagent in combination with electroporation has been employed to study brain tumor model (98). Viral vector-based transfections have also been attempted *in vivo*. For example, AAV delivery has been used to study lung carcinogenesis by applying them intra-tracheally *in vivo* (96). Furthermore, lentiviral-based constructs were used in a trial involving a pancreatic ductal adenocarcinoma mouse model (142).

Although the above review of various delivery methods that have already been attempted in transgenic mouse models of cancer demonstrates some degree of initial success, several challenges remain to be solved. One such challenge is to enable the delivery of Cas9 ribonucleoprotein complexes and donor DNA *in vivo* to induce homology-directed DNA repair and repair cancer-causing mutations. A very recent study by Lee et al. (143) used gold nanoparticles conjugated to DNA and complexed with cationic endosomal disruptive polymers, and the results demonstrated correction of the DNA mutation that causes Duchenne muscular dystrophy in mice. Such an approach should be of interest for mouse cancer models, especially inherited forms of driver mutations. Finally, improvements in delivery methods to increase specificity and efficiency and to minimize off-target events and immune response are necessary to ensure the validity of mouse cancer models and to increase their translational potential.

### Pitfalls and Limitations

As with every novel technology, there are pitfalls and limitations that must be overcome using the CRISPR/Cas9 system in the future. For example, in modeling small deletions and insertions, current CRISPR/Cas9-based gene editing uses NHEJ-mediated mechanisms that generate small indels, but the sequence variation in the generated allelic series is enormous. While indels usually generate loss-of-function alleles, certain indels can be in-frame or out-of-frame, generating truncated or modified gene products with different phenotypic effects. Apart from the aforementioned loss-of-function models, a greater challenge is still the development of precise cancer-driver mutations *in vivo* by the HDR mechanism. This approach continues to have room for improvements to efficiently generate gain-of-function mutations that are prevalent in carcinogenesis. As mentioned earlier, the CRISPR/Cas9 system allows multiplexing and hence sequential mutagenesis of cancer genes to model loss- or gain-of-function events that are frequently found in human cancer genomes.

The off-target editing activity of the CRISPR/Cas9 system presents a concern and potential limitation. This activity could affect the phenotype of CRISPR/Cas9-generated mouse mutants, such that the phenotype is not related to the on-target event but rather some modification(s) elsewhere in the genome. While some studies in human cells report a relatively high frequency of off-target events (144), early data in mouse embryos suggest that CRISPR/Cas9 off-target events are very rare (89, 145). To examine in detail the extent of off-target events, next-generation whole-genome sequencing has recently been used. Such studies now show that the likelihood of off-target events can be minimized by the careful design of guide RNAs and selection of genomic target sites (146). This result is further supported in a large-scale screen for off-target events in CRISPR/Cas9 transgenic mice performed by Singh et al. (105). For gRNAs selected to have low off-target hit scores, 90 founder mice were screened in 56 of the highest-scoring off-target sites, but no cases of off-target mutagenesis were recorded. To further minimize the off-target activity of Cas9, which will especially be important in eventual human therapy, researchers have attempted to modify the Cas9 protein itself, by using a truncated gRNA or by a method of “paired nicks” (147, 148). Direct use of recombinant Cas9 protein can also lower the off-target editing frequency, most likely because Cas9 protein degrades much faster once it is in the cell than the plasmid encoding Cas9 (149). Although some recent studies report advances in minimizing off-target effects (150), both future preclinical and especially clinical applications will require essentially no detectable genome-wide off-target activity. Developments in the area of high-throughput genome-wide sequencing will certainly aid in allowing the efficient identification of such off-target effects (151) and should be routinely used in future cancer model studies.

Another area that will most likely gain more attention is the combination of conventional cancer models with CRISPR/Cas9 tools to edit genes and simultaneously affect gene expression without any genome editing. Such orthogonal approaches for using the nuclease activity-deficient dCas9-effector system in combination with the editing Cas9-based system should soon be more frequently applied in mouse models of cancer. Namely, Cas9 variants isolated from different bacterial species (152, 153) or mutated forms of Cas9 from the same species that recognize different PAM sites next to the sgRNA-binding site (154) are now available. Such combinatorial approaches can be used to generate more complex mouse models of human cancers, which is certainly a complex disease.

## Conclusion

Traditional mouse cancer models have already contributed immensely toward illuminating the mechanistic underpinnings of carcinogenesis and will continue to be used on their own or in combination with more recently developed models. One criticism regarding the use of traditional mouse transgenic models lies in their limitations with respect to the model design and relatively slow translational potential for more rapid and improved benefits for cancer patients.

In recent years, new mouse models of human cancer were developed that may overcome these limitations by accelerating the detection of novel cancer genes, deciphering mechanisms of carcinogenesis, establishing more relevant mouse cancer models, and examining novel approaches to cancer treatments to obtain the maximum value for cancer patients. We envisage that future developments and applications in mouse transgenic cancer modeling will be focused primarily in two areas. One such area of current and future intense research will be concentrated on the use of the CRISPR/Cas9 system as the most versatile and adaptable transgenic technology to date producing transgenic mice that resemble the exact steps of human carcinogenesis. The sequence data for an individual patient tumor, which can now be obtained in a more cost-effective way, can be functionally validated using CRISPR/Cas9 transgenic *in vitro* and *in vivo* mouse models. Thus, all the improvements and results from these novel mouse cancer models will hopefully help to reveal more genotype-specific susceptibilities of particular human cancer types to finally enable more personalized, genotype-based treatments for cancer patients.

Conversely, since increasingly more is known about the importance of the tumor microenvironment, not only on tumor growth but also on the local and systemic response to therapy, there is a more extensive demand for the development of mouse models that more accurately represent the human tumor microenvironment. Humanized mouse models with implanted PDX and human microbiota would bring cancer immunotherapy research one step further, enabling the examination of the complex interaction between the tumor, immune system, and microbiome as one system in the patient. This approach could potentially be used to screen for effective immunotherapeutic agents or combinations, to study mechanisms of resistance to immunotherapies and to study approaches on how to turn immunologically cold tumors into hot ones. Although conceptually diverse, both applications have the final aim to tailor therapeutic regimens based on specific molecular profiles of tumors. The majority of the applications of these two approaches are still at the preclinical stage, but they show great promise to soon become more clinically relevant as they develop toward a more mature stage.

Taken together, forthcoming improvements in mouse cancer models might present one successful pathway to precise individualized cancer therapy, leading to improved cancer patient survival and quality of life.

## Author Contributions

All authors were involved with the conception and design of the manuscript, manuscript writing, and final approval of the manuscript.

## Conflict of Interest Statement

The authors declare that the research was conducted in the absence of any commercial or financial relationships that could be construed as a potential conflict of interest.
